# HuD Binds to and Regulates Circular RNAs Derived From Neuronal Development- and Synaptic Plasticity-Associated Genes

**DOI:** 10.3389/fgene.2020.00790

**Published:** 2020-08-05

**Authors:** Michela Dell’Orco, Robert J. Oliver, Nora Perrone-Bizzozero

**Affiliations:** Department of Neurosciences, School of Medicine, University of New Mexico Health Sciences Center, Albuquerque, NM, United States

**Keywords:** RNA-binding protein, neuronal differentiation, posttranscriptional regulation, synaptic plasticity, circular RNA, conditioned place preference (CPP), HuD, ELAVL4

## Abstract

The RNA-binding protein (RBP) HuD is involved in neuronal differentiation, regeneration, synaptic plasticity and learning and memory. RBPs not only bind to mRNAs but also interact with several types of RNAs including circular RNAs (circRNAs), a class of non-coding RNAs generated by pre-mRNA back-splicing. This study explored whether HuD could regulate the expression of neuronal circRNAs. HuD controls target RNA’s fate by binding to Adenylate-Uridylate Rich Elements (AREs). Using bioinformatics analyses, we found HuD-binding ARE-motifs in about 26% of brain-expressed circRNAs. By RNA immunoprecipitation (RIP) from the mouse striatum followed by circRNA arrays, we identified over 600 circRNAs bound to HuD. Among these, 226 derived from genes where HuD also bound to their associated mRNAs including *circHomer1a*, which we previously characterized as a synaptic HuD target circRNA. Binding of HuD to two additional plasticity–associated circRNAs, *circCreb1*, and *circUfp2*, was validated by circRNA-specific qRT-PCR. Interestingly, we found that *circUpf2* is also enriched in synaptosomes. Pathway analyses confirmed that the majority of HuD-bound circRNAs originate from genes regulating nervous system development and function. Using striatal tissues from HuD overexpressor (HuD-OE) and knock out (KO) mice for circRNA expression analyses we identified 86 HuD-regulated circRNAs. These derived from genes within the same biological pathways as the HuD RIP. Cross-correlation analyses of HuD-regulated and HuD-bound circRNAs identified 69 regulated in either HuD-OE or HuD-KO and 5 in both sets. These include *circBrwd1*, *circFoxp1*, and *circMap1a*, which derive from genes involved in neuronal development and regeneration, and *circMagi1* and *circLppr4*, originating from genes controlling synapse formation and linked to psychiatric disorders. These circRNAs form competing endogenous RNA (ceRNA) networks including microRNAs and mRNAs. Among the HuD target circRNAs, *circBrwd1* and *circFoxp1* are regulated in an opposite manner to their respective mRNAs. The expressions of other development- and plasticity-associated HuD target circRNAs such as *circSatb2, cirHomer1a* and *circNtrk3* are also altered after the establishment of cocaine conditioned place preference (CPP). Collectively, these data suggest that HuD interactions with circRNAs regulate their expression and transport, and that the ensuing changes in HuD-regulated ceRNA networks could control neuronal differentiation and synaptic plasticity.

## Introduction

The neuronal RNA-binding protein (RBP) HuD (aka, ELAVL4) is involved in multiple steps of neuronal differentiation and maturation from neurogenesis to axonal and dendritic outgrowth and remodeling (Perrone-Bizzozero and Bolognani, 2002; Bolognani et al., 2004, 2007; Oliver et al., 2018). This protein is a member of the Hu protein family, which are orthologs of Drosophila ELAV (Embryonic Lethal Abnormal Vision) (Campos et al., 1985). HuD controls nervous system development and function by post-transcriptional regulating mRNAs from proteins associated with neuronal differentiation and synaptic plasticity such as SATB1, GAP-43, BDNF, CAMKIIα, and HOMER1 (Tiruchinapalli et al., 2008; Bolognani et al., 2010; Allen et al., 2013; Sosanya et al., 2015; Wang et al., 2015). Changes in gene expression have also been linked to the mechanisms of learning and memory (Sweatt, 2016) and HuD levels were found to increase in both the mouse and rat hippocampus during associative- and spatial-learning and memory paradigms (Bolognani et al., 2004; Pascale et al., 2004; Bolognani et al., 2007). HuD controls the stability of target mRNAs by binding to Adenylate-Uridylate Rich Elements (AREs) in the 3′ untranslated regions (3′ UTRs) via the first two RNA recognition motifs (RRMs) and to the poly(A) tails via its third RRM (Wang and Tanaka Hall, 2001; Beckel-Mitchener et al., 2002; Pascale et al., 2005; Bolognani et al., 2010; Sosanya et al., 2013). However, whether HuD could bind to similar sequences in non-coding RNAs (ncRNAs) was not fully investigated until now.

RBPs, including HuD, not only bind to mRNAs but also interact with other types of RNAs in the cell including circular RNAs (circRNAs), a novel class of ncRNAs generated by back-splicing of pre-mRNAs and some long ncRNAs (lncRNAs) (Wilusz, 2016; Li B. et al., 2017). CircRNAs are particularly enriched in the brain, where they are more stable and regulated independently from their linear counterparts (Rybak-Wolf et al., 2015; You et al., 2015). Neuronal circRNAs derive from genes encoding proteins involved in synaptic formation and plasticity and more enriched in synapses than their linear isoforms (You et al., 2015; Chen and Schuman, 2016). About 58% of CNS circRNAs are upregulated throughout development, particularly during synaptogenesis, with levels increasing from embryonic to the adult brain (Mahmoudi and Cairns, 2019). Recent studies show accumulation of circRNAs with normal aging and their dysregulation in neurodegenerative diseases and other neuropsychiatric disorders (Wang et al., 2018; Cai et al., 2019; Dube et al., 2019; Mahmoudi and Cairns, 2019; Mehta et al., 2020; Zimmerman et al., 2020).

There are different subtypes of circRNAs including exonic, intronic and exo-intronic (Xie et al., 2017). Exonic circRNAs are mostly localized in the cytoplasm where they are thought to act as decoys or “sponges” for RBPs or microRNAs (miRNAs). Thus, one possibility is that they inhibit the interaction of RBPs and miRNAs with their target mRNAs (Kristensen et al., 2019). Several RBPs have been identified as regulators of circRNA biogenesis including ADAR1 (Ivanov et al., 2015; Rybak-Wolf et al., 2015), QKI (Conn et al., 2015), FUS (Errichelli et al., 2017), MBL (Ashwal-Fluss et al., 2014), HNRNPL (Fei et al., 2017), SRs (Kramer et al., 2015), NF90 and NF110 (Li X. et al., 2017). Interestingly, not only is the expression of multiple RBPs spatiotemporally regulated similarly to circRNAs, but also RBPs such as the ELAV-like protein HuR have been shown to bind to circRNAs, which can sequester them blocking their function (Dudekula et al., 2016; Abdelmohsen et al., 2017).

We have recently shown that HuD binds to and regulates the synaptic localization of *circHomer1a* (Zimmerman et al., 2020). To define the global role of this RBP in circRNA regulation, in this study, we sought to identify mouse circRNAs that are bound and regulated by HuD. As in previous studies examining circRNA-protein interactions (Abdelmohsen et al., 2017), we used HuD RNA immunoprecipitation (RIP) coupled with circRNA arrays to identify circRNAs bound to HuD *in vivo*. We also used HuD overexpressing mice and HuD-KO to evaluate whether changes in HuD levels could alter target circRNA expression. Finally, we assessed the relationship of HuD and its target circRNAs during cocaine conditioned place preference (CPP), a model of addiction-related behaviors. Our results demonstrate that HuD binds and regulates the levels of multiple circRNAs from genes involved in neuronal differentiation, synapse formation and learning and memory and that the expression of several of these circRNAs is altered during cocaine CPP.

## Materials and Methods

### Bioinformatics Analysis

Analysis of ARE sequences was performed using previously published scripts (Bolognani et al., 2010). Besides single-stranded sequences, this specific analysis considers restrictions imposed by the three dimensional complex between the first two RNA Recognition Motifs (RRMs) in HuD and two different Class I and II AREs (Wang and Tanaka Hall, 2001; Bolognani et al., 2010). All mouse circRNAs sequences were downloaded from circBase^1^ (Glažar et al., 2014).

### HuD-OE and KO Mice

HuD-KO (Elavl4^–/–^) mice were a gift from Prof. Hideyuki Okano (Akamatsu et al., 2005). Transgenic mice overexpressing myc-tagged HuD under the Camk2a promoter were generated and backcrossed to a C57BL/6J background (Bolognani et al., 2006, 2007). All experimental procedures were performed in accordance with the National Institutes of Health Guide for Care and Use of Laboratory Animals and were approved by the University of New Mexico Health Sciences Center Institutional Animal Care and Use Committee.

### RNA Immunoprecipitation

circRNAs bound to HuD were isolated from the striatum of HuD-OE mice by RNA immunoprecipitation (RIP) using Dynabeads^®^ (Thermo Fisher Scientific) coated with mouse monoclonal anti-myc tag antibody (9B11; Cell Signaling Technology Inc.) specifically recognizing myc-tagged HuD transgenic protein expressed in HuD-OE as described before (Bolognani et al., 2010; Zimmerman et al., 2020). Controls for RIP assays were performed using either non-immune IgG and HuD-OE tissue or the myc-tag antibody and wild type (WT) tissue. Aliquots for both RNA and proteins were taken before (input) and after the IP and circRNAs were quantitated using circRNAs arrays as described below. Immunoprecipitated RNA was extracted with Trizol^®^ (Invitrogen) according to manufacturer instructions and subjected to either circRNA array or qRT-PCR as described below.

### RNA Extractions From HuD-OE and KO Mice

Total RNA was isolated from the striatum of HuD-OE, HuD KO and wild type mice using Trizol^®^ (Invitrogen) as described above. RNA quality and quantity was determined using the Qubit (Invitrogen) spectrophotometer. Aliquots of the same RNA were used for circRNA and mRNA arrays as described below.

### circRNA Arrays

Sample preparation and microarray hybridization were performed by Arraystar, Inc. Briefly, total RNAs were digested with RNAse R (Epicentre, Inc.) to remove linear RNAs. Then, circRNAs were amplified and transcribed into fluorescent cRNA utilizing a random priming method (Super RNA Labeling Kit; Arraystar) and labeled cRNAs hybridized onto the Arraystar Mouse circRNA Array V2 (8 × 15 K, Arraystar).

### mRNA Arrays

The sample preparation and microarray hybridization were performed based on the Arraystar’s standard protocols described above. The labeled RNAs were hybridized onto the Mouse LncRNA and mRNA Arrays v3.0 (8 × 60 K, Agilent).

### Synaptosome Purification

Crude synaptosomes were prepared from the mouse stratum as previously described (Rao and Steward, 1991; Boese et al., 2016). Briefly, the tissue was homogenized in 500 μl of ice cold homogenization buffer (0.32 M sucrose, 0.1 mM EDTA, 0.25 mM DTT, 2 mM HEPES, pH 7.4) supplemented with 200 U/ml RNaseOUT^TM^ (Invitrogen). Nuclei and cell debris were removed by centrifugation for 2 min at 2000 × *g* and the supernatant (S1) was centrifuged for additional 10 min at 14,000 × *g*. The second supernatant (S2) was collected and the resulting crude synaptosomal pellet was used for total RNA extraction with Trizol^®^ (Invitrogen) as described above. To test purification efficiency, levels of the small brain specific RNA BC1, which is enriched in synaptosomes (SYN) (Boese et al., 2016), were determined in the S1 and S2 supernatant fractions and the final pellet.

### Western Blot Analysis

Immunoprecipitated proteins or input proteins (5 μg) were separated on 10% SDS–polyacrylamide gels and transferred onto a PVDF membrane (Bio-Rad) as described before (Tanner et al., 2008; Oliver et al., 2018). The membranes were treated with a blocking solution containing 5% non-fat dry milk in TBS-T buffer (10 mM Tris–HCl, 100 mM NaCl, 0.1% Tween, pH 7.5) and incubated overnight with mouse monoclonal anti-HuD (E1, sc-28299; Santa Cruz Biotechnology, Inc.; dilution 1:10000 in blocking solution). Blots were then incubated using secondary, anti-mouse peroxidase-conjugated antibodies (GE Healthcare, United States, dilution 1:10,000) and immunoreactive bands detected using the Western Lightning Plus-ECL (PerkinElmer Inc., United States).

### circRNA-Specific qRT-PCR

Divergent primers were designed with NCBI PrimerBlast (Ye et al., 2012) to amplify sequences surrounding the circRNA specific back-splicing junction (BSJ). Primer validation and qRT-PCR for circRNAs was performed using random primers as previously described (Panda and Gorospe, 2018; Zimmerman et al., 2020).

### Ingenuity Pathway Analysis

Molecular functions and cellular pathways of genes hosting circRNAs regulated by HuD were identified using Ingenuity Pathway software (IPA, Winter 2019 Release, Qiagen^2^).

### ceRNA Analysis

ceRNA analyses were conducted by Arraystar, Inc., as previously described (Shao et al., 2018; Wang et al., 2018). circRNA/miRNA/mRNA networks were generated to visualize ceRNA interactions.

### Cocaine Conditioned Place Preference

For cocaine CPP, 2 month old Male C57Bl/6J mice underwent training as previously described (Allan et al., 2001; Oliver et al., 2018). Mice were sacrificed 1 h after testing and the nucleus accumbens (NAc) was collected. Total RNA was extracted with Trizol^®^ (Invitrogen) and circRNAs analyzed using Arraystar arrays as described above.

### Statistical Analysis

All experiments were performed in triplicates, using sample numbers as indicated in each figure legend. Values were expressed as means ± SEM. Statistical analysis was performed using GraphPad Prism version 8 (La Jolla, CA, United States). The data were analyzed by Student *t*-tests or a One-way analysis of variance (ANOVA) followed by Multiple Comparison Tests. Differences were considered statistically significant when *p*-values were < 0.05.

## Results

### HuD Binds to circRNAs From Genes Regulating Nervous System Development and Function

**FIGURE 1:**
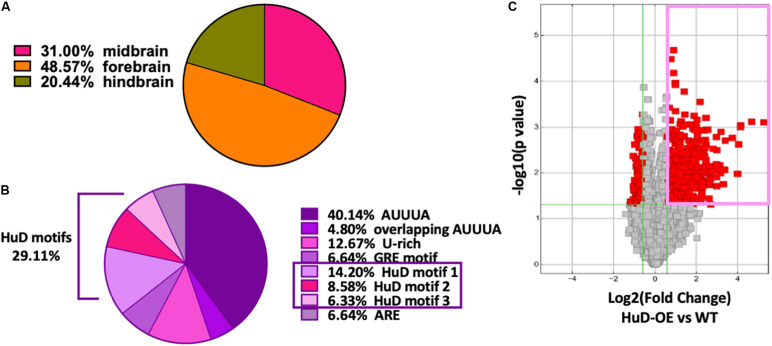
High frequency of HuD binding motifs in mouse brain circRNAs. **(A)** 38.7% of mmu_circRNAs are expressed in the brain; of these, 31% in the midbrain, 48.57% in the forebrain and 20.44% in the hindbrain. **(B)** Bioinformatics analysis of mouse circRNAs showing distribution of ARE sequences. Completely annotated mmu_circRNA sequences from circBase were analyzed and 33 % were found to have HuD consensus binding motifs and other ARE motifs. Pie chart from this set of circRNAs shows the percentage of each ARE motif including 29.11% of HuD specific motifs (14.20% motif 1; 8.58% motif 2, 6.33% motif 3). **(C)** Volcano plots representing the differential enrichment of circRNAs in the HuD RIP vs. WT controls (pink rectangle). The RNA obtained after HuD immunoprecipitation was subjected to circRNA array analysis as described in section “Materials and Methods.” The vertical lines correspond to 1.5-fold change up and down, respectively, and the horizontal line represents a *p*-value of 0.05, *n* = 3 for each IP (HuD-OE, WT and IgG); three mice were pulled together for each IP and the tissue analyzed in three separate RIP assays. Data points in red represent differentially expressed circRNAs.

Thousands of circRNAs are known to be expressed in mouse tissues (Rybak-Wolf et al., 2015) and their complete sequences are available at circBase database (Glažar et al., 2014). Remarkably, 38.7% of all mmu_circRNAs are expressed in the brain: of these, 31% in the midbrain, 48.57% in the forebrain and 20.44% in the hindbrain (Figure 1A). Using previously published Perl scripts developed in our lab (Bolognani et al., 2010), we performed bioinformatics analyses of all mouse circRNAs (mmu_circRNAs) to identify HuD-binding consensus motifs and other ARE sequences. We found that about 33% of mmu_circRNAs contain different ARE motifs and, of these, 29.11% bear HuD motifs 1, 2, or 3 (Figure 1B). Considering that HuD also interacts with other ARE and GU-rich elements (GRE), the percent of circRNAs with HuD binding motifs could reach up to 42% of all ARE containing circRNAs (Figure 1B). Overall, the predicted set of HuD-interacting circRNAs represents about 10% of the total set of mouse circRNAs and 26% of all brain-expressed circRNAs. In comparison, about 20% of all neuronal mRNAs in the adult mouse forebrain contain ARE sequences for interactions with HuD and other ARE-binding proteins (Bolognani et al., 2010), suggesting that circRNAs and mRNAs may share similar mechanisms of post-transcriptional regulation.

**FIGURE 2:**
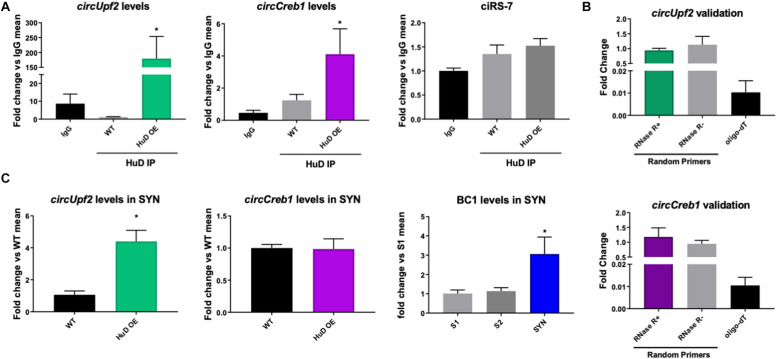
HuD binds to circUpf2 and circCreb1 in vivo. **(A)** The interaction between HuD and the circRNAs derived from the Upf2 and Creb1 genes was confirmed by HuD RIP vs. WT control RIP followed by circRNA-specific qRT-PCR assays. Values are expressed as fold change vs. IgG controls. Mmu_circ_CiS-R7 was used as negative control. Data were analyzed by a One-way ANOVA, followed by Dunn’s Multiple Comparison Test and expressed as mean ± SEM; *p < 0.05, *n* = 3. **(B)** Validation of specific amplification of circRNAs by qRT-PCR using RNAse R to pretreat the RNA to degrade all linear RNAs and random primers vs. oligodT to amplify circRNAs vs. mRNAs. *n* = 3. **(C)** Total RNA from synaptosomes extracts from the mouse striatum were processed as described in section “Materials and Methods” and subjected to qRT-PCR for circUpf2 and circCreb1. Values were normalized to GAPDH mRNA levels and expressed as fold change vs. WT controls; BC1 RNA levels differences between S1, S2 and synaptosomes fraction (SYN) were used to validate synaptosome (SYN) purification as previously described (Rao and Steward, 1991). Data was analyzed by Student *t*-tests (circUpf2 and circCreb1) and One-way ANOVA (BC1). Values represent mean ± SEM; *p < 0.05, *n* = 3.

To test whether HuD binds to circRNAs *in vivo*, we performed RNA immunoprecipitation (RIP) from the striatum of HuD-OE mice (Figure 1C) using a myc-tag antibody specific to the transgenic protein. RIP with non-immune IgG or WT mouse tissue were used as controls. Using mouse circRNAs arrays, we identified 670 circRNAs interacting with HuD [fold-change (FC) > 1.5, *p* < 0.05], which were derived from 500 different genes. The fold change cutoff was selected using the level of transgene expression in the striatum (Oliver et al., 2018). HuD-bound circRNAs, represented in red symbols inside the pink rectangle in Figure 1C, are listed in Supplementary Table 1. Among these is *circHomer1a*, a plasticity-associated HuD target circRNA that we previously identified using RIP and qRT-PCR (Zimmerman et al., 2020). Here, we also used circRNA-specific qRT-PCR assays to validate binding of HuD to two additional circRNAs from plasticity-associated protein-coding genes: *circUpf2* and *circCreb1* (Figure 2A). As a negative control we used ciRS-7 (aka, *Cdr1as*, Hansen et al., 2013), since it does not have any Hu protein binding sequences (Abdelmohsen et al., 2017). HuD binding to *circUpf2* and *circCreb1* is significantly increased in the HuD-RIP compared to both WT and IgG negative controls (Figure 2A). The specificity of qRT-PCR reactions was confirmed by the lack of sensitivity to pretreatment of RNA with RNase R, which degrades all linear RNAs, and the more than 100-fold amplification with random primers relative to oligo-dT (Figure 2B). Both circRNAs contain multiple HuD binding ARE motifs in their sequences (Supplementary Figure 1). Considering the localization of circRNAs and HuD in synapses, we also tested whether HuD binding could lead to increased levels of target circRNAs in synaptosomes (SYN). The levels of the synaptically enriched BC1 RNA confirmed the yield of synaptosomes after purification (Boese et al., 2016). As we have shown previously for *circHomer1a* (Zimmerman et al., 2020), we found that *circUpf2* levels increased 4-fold in synaptosomes from HuD-OE mice compared to WT. However, no changes were observed in *circCreb1* (Figure 2C).

**FIGURE 3:**
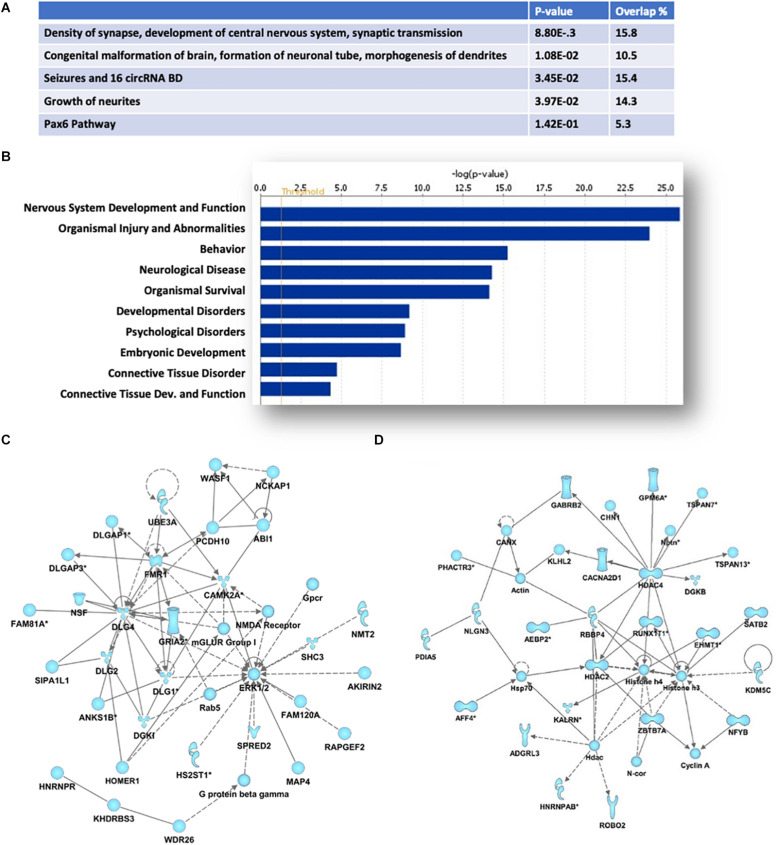
Pathway and functional analyses of host genes of HuD target circRNAs. Ingenuity Pathway Analysis of HuD interacting circRNAs identified by RIP. **(A)** Top molecular functions associated with genes associated with HuD-bound circRNAs. **(B)** Top Diseases and Functions. **(C,D)** Representative networks including circRNAs from genes encoding proteins involved in glutamatergic transmission **(C)** and epigenetic regulators **(D)**.

Ingenuity Pathway analyses of the protein-coding genes associated with the 670 HuD-interacting circRNAs showed an enrichment in those regulating cellular development, growth and proliferation and morphology (Figure 3A). Moreover, the majority of the host genes of the circRNAs bound by HuD are associated to nervous system development and function, behavior and neurological diseases (Figure 3B). As shown in Figures 3C,D, the top two biological networks from HuD-bound circRNAs derive from genes regulating cell signaling, cell morphology and cell-to-cell interactions. These include genes encoding proteins involved in glutamatergic transmission such as FMR1, CAMK2A, GRIA2, mGluRI, HOMER1, and NMDA receptor (NMDAR) subunits (Figure 3C) and those controlling neuronal development and synapse formation (ROBO2, NLGN3) as well as GABAergic transmission (GABRB2) and epigenetic regulators such as SATB2 and histone deacetylases (HDAC2, HDAC4) (Figure 3D). Additional networks and their associated genes are shown in Supplementary Table 2. Interestingly, 226 of the host genes of HuD-bound circRNAs, generate mRNAs that also bind to HuD, indicating that this RBP could be regulating both circular and linear RNAs derived from the same genes (Supplementary Table 3).

### HuD Regulated circRNAs Form Competing Endogenous RNAs (ceRNAs) Networks

**FIGURE 4:**
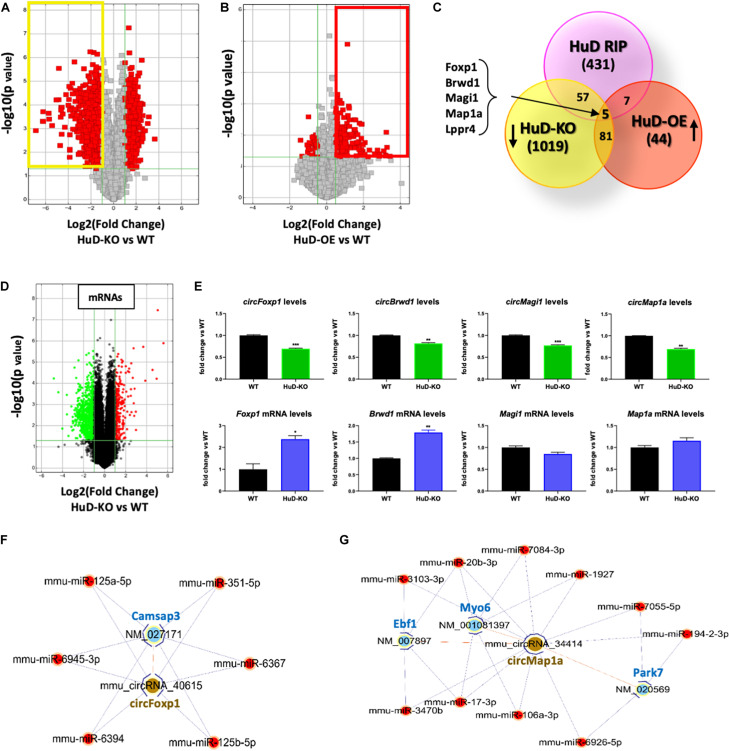
Profiling of circRNA expression changes in HuD-OE and HuD-KO mice. **(A)** Volcano plots representing circRNAs differentially expressed between HuD-KO (*Elavl4^–^^/^****^–^***) mice and WT littermates. The vertical lines correspond to 2-fold change up and down, respectively, and the horizontal line represents a *p*-value of 0.05. Values shown in red represent the differentially expressed circRNAs. *n* = 3 for each genotype (HuD-KO, WT). **(B)** Volcano Plots representing circRNAs differentially expressed between HuD-OE mice and WT littermates. RNA extracted from HuD-OE and WT mouse striatum was subjected to circRNA array as described in Methods. The vertical lines correspond to 1.5-fold change up and down, respectively, and the horizontal line represents a *p*-value of 0.05. Values shown in red represent the differentially expressed circRNAs. *n* = 3 for each genotype (HuD-OE, WT) **(C)** Identification of common elements between circRNAs bound to HuD and circRNAs whose expression is regulated by HuD. A list of 5 candidate HuD circRNAs for validation was obtained comparing the results of the three different arrays. **(D,E)** Comparison of circular and linear RNA levels. **(D)** RNA extracted from HuD-KO and WT mice striatum was subjected to mRNA microarray analysis as described in section “Materials and Methods.” The vertical lines in the volcano plot correspond to 2-fold change up or down, respectively, and the horizontal line represents a *p*-value of 0.05. Values shown in red or green represent the differentially up- and down-regulated mRNAs. *n* = 3 for each genotype. (HuD-KO, WT). **(E)** Graphs showing levels of both circular **(top)** and linear **(bottom)** RNA levels in HuD-KO mice vs. WT. Data are Means ± SEM, *p < 0.05, Student’s *t*-test. **(F,G)** ceRNA networks. ceRNA analyses were performed as described in sction “Materials and Methods.” circRNA/miRNA/mRNA networks were generated to visualize the interactions for *circFoxp1* and *circMap1*; the ceRNAs of the remaining circRNAs are reported in Supplementary Table 3.

To further understand the biological significance of HuD-circRNA interactions, we investigated the expression of circRNAs in the striatum of HuD-KO and HuD-OE mice. CircRNAs arrays identified 1162 circRNAs significantly downregulated in HuD-KO mice (FC > 2, *p* < 0.05; Figure 4A, yellow rectangle) and 137 circRNAs significantly upregulated in HuD-OE mice (FC > 1.5, *p* < 0.05 Figure 4B; red rectangle). Cross-correlation analyses of HuD-regulated and HuD-bound circRNAs identified 86 circRNAs upregulated in HuD-OE and downregulated in HuD KO and 69 regulated in either HuD-OE or HuD-KO and bound to HuD. Finally, five circRNAs were found to be present in all three datasets (Figure 4C). These include *circBrwd1*, *circFoxp1*, *circMap1a*, *circMagi1*, and *circLppr4*, which derive from genes regulating neuronal development, regeneration and synaptic plasticity. The complete list of the circRNAs identified by cross-correlation analyses is shown in Supplementary Table 4.

Additional mRNA microarray analyses from the striatum of HuD-KO mice (Figure 4D) identified 298 upregulated and 790 downregulated transcripts. Comparison of circRNA and mRNA levels from the same genes reveal that the decreases in *circFoxp1* and c*ircBrwd1* in HuD KO tissues are associated with increased levels of their respective mRNAs. In contrast, no changes in mRNA levels were associated with decreases in *circMap1a* and *circMagi1* levels (Figure 4E). These results suggest that other factors could be involved in the regulation of circRNA vs. mRNA levels by HuD. Competing Endogenous RNAs (ceRNAs) analyses enabled us to identify miRNAs interacting with the circRNAs targeted by HuD and mRNAs targeted by the same miRNA sequences (Supplementary Table 5). Visual representations of ceRNA networks for *circFoxp1* and *circMap1* are shown in Figures 4F,G. *circFoxp1* interacts with the mRNA for *Camsap3* (calmodulin-regulated spectrin-associated protein 3 Also, *circFoxp1* and *Camsap3* interact with several miRNAs (Figure 4F) including miR-125a-5p, which has been previously associated with aging (Makwana et al., 2017). *circMap1a* interacts with *Ebf1*, which encodes a protein involved in cell differentiation in embryonic striatum (Garel et al., 1999) and the mRNAs for synaptic transmission- and neuroprotection-associated genes, *Myo6* and *Park7* (Figure 4G). A ceRNA network for *circBrwd1* is shown in Supplementary Figure 2.

### Multiple HuD Target circRNAs Are Regulated During Addiction-Related Behaviors

**FIGURE 5:**
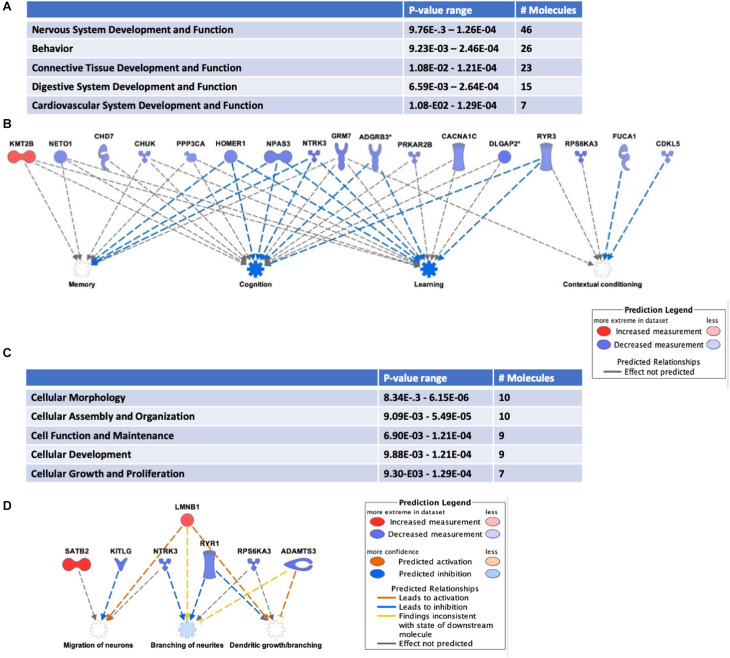
Cocaine CPP alters the expression of circRNAs from behavior-associated genes. RNA extracted from the NAc of mice after CPP testing was subjected to circRNAs arrays as described in section “Materials and Methods.” **(A)** Differentially expressed circRNAs were analyzed to identify Top Diseases and Functions (FC > 1.2, *p* < 0.05). *n* = 3 for each condition (Saline control, Cocaine). **(B)** Representation of molecules and functions from behavior-associated circRNAs. Molecules in blue are downregulated and in red are upregulated. Blue arrows indicate the molecule is inhibiting the pathway and gray arrows indicate the lack of information on the pathway interaction. **(C)** Molecular functions associated with top circRNA changes (FC > 1.3, *p* < 0.05). **(D)** Representation of molecules and functions from the Cellular Growth and Proliferation-associated circRNAs in panel **(C)**. Molecules in blue are downregulated, in red are upregulated; color intensity is proportional to the fold change in expression levels after CPP. Blue arrows indicate the molecule is inhibiting the pathway, while red arrows indicate activation.

Addiction-associated synaptic plasticity involves the simultaneous influence of mRNA post-transcriptional regulation and protein translation. Therefore, it is not surprising that HuD has been identified as a regulator of many addiction-related genes (Oliver et al., 2018). Conditioned placed preference (CPP) to drugs of abuse such as cocaine is a model of addiction-related behaviors used to assess the reinforcing effect of drugs (Tzschentke, 2007; Huston et al., 2013). HuD levels are upregulated in nucleus accumbens (NAc) after cocaine CPP. Therefore, to investigate if any of the HuD-bound circRNAs was also regulated in this behavioral paradigm, we used RNA derived from the same tissues to measure circRNA expression in cocaine CPP vs. saline control mice. Among the circRNAs whose levels were significantly altered by CPP (see Supplementary Table 6), eight of them (*circNpas3*, *circCdk17*, *circHomer1a*, *circNtrk3*, *circSatb2*, *circTomm70a*, *circRab30*, and *circDnm1*) are HuD targets identified by our HuD RIP studies (Supplementary Table 1) and six (*circAhcyl2*, *circDlgap2*, *circLmo7*, *circRps6ka3*, *circTrmt2b*, and *circXkr6*) have HuD binding motifs (Bolognani et al., 2010) and thus, are predicted to bind HuD. For example, HuD binding sequences are shown for *circSatb2* (see Supplementary Table 6). Pathway analyses revealed that the majority of circRNAs whose levels were significantly upregulated or downregulated (FC > 1.2 or < 0.80, *p* < 0.05) by cocaine CPP derive from genes involved in nervous system development (46 molecules), behavior (26 molecules) (Figure 5A) as well as other nervous system-specific pathways (Supplementary Table 7). The genes associated with behavior encode HOMER1, NTRK3 (TrkC) and NPAS3, all proteins that regulate cognition, learning and memory (Figure 5B). A complete list of circRNAs derived from genes associated with behavior is shown in Supplementary Table 8. Furthermore, the top 30 most changed circRNAs are specifically involved in pathways associated with the establishment of synaptic, dendritic and axonal morphology: Cellular Assembly and Organization; Cellular Function and Maintenance and Cellular Growth and Proliferation (Figure 5C). The network in Figure 5D shows circRNAs both upregulated, e.g., *circSatb2* (red), and downregulated, e.g., *circNtrk3* (blue), by cocaine CPP. These molecules control migration of neurons, branching of neurites, and dendritic growth and branching. In addition, we discovered a potential bidirectional regulation of these processes, with molecules inhibiting (blue arrows) and molecules activating them (red arrows).

## Discussion

CircRNAs are highly abundant in the brain, where they are particularly enriched in synapses and upregulated during synaptic formation and plasticity (Rybak-Wolf et al., 2015; You et al., 2015). Given our recent finding that the neuronal RBP HuD binds to and regulates the synaptic localization of one of these synaptic plasticity-associated circRNAs, *circHomer1a* (Zimmerman et al., 2020), in this study we sought to globally identify mouse circRNAs that are bound and regulated by this RBP. Bioinformatics analyses of HuD binding sites revealed consensus HuD binding ARE motifs in about 26% of brain-expressed circRNAs, which is comparable to our previous finding of these sequences in 20% of forebrain expressed mRNAs (Bolognani et al., 2010). Using a combination of RNA-IP and circRNA array analyses as well as qRT-PCR validations, we confirmed HuD binding to striatal circRNAs from genes involved in neuronal differentiation, synapse formation and learning and memory. Furthermore, the levels of multiple circRNAs within these pathways also depend on the levels of HuD as demonstrated by their changes in HuD-OE and KO mouse tissue. A few of these circRNAs are predicted to interact with miRNAs and mRNAs forming ceRNA networks. Finally, we found that the expression of several HuD target circRNAs is also altered during cocaine CPP.

Many of the HuD interacting circRNAs identified here derive from genes that control dendrite morphogenesis, synapse density and synaptic transmission. Among the HuD target circRNAs, are *circCREB1* and *circUpf2*. *CircCreb1* derives from the gene encoding the well-characterized activity-depended transcription factor CREB1, which is involved in learning and memory and addiction (Kandel, 2012; Nestler, 2012). Also, *circUpf2* is of particular interest since previous studies demonstrated that *in vivo* deletion of *Upf2* gene, one of the components of nonsense mediated decay (NMD), in adult glutamatergic neurons alters spine density and LTP in the adult hippocampus and disrupts learning and memory (Notaras et al., 2019). Our findings that *circUpf2* is enriched in synaptosomes from HuD-OE mice suggest that this circRNA may also regulate synaptic plasticity. Our previous observation that the synaptic localization of another HuD target circRNA, *circHomer1a*, alters the synaptic localization of *Homer1b* mRNA and reversal learning (Zimmerman et al., 2020), suggests that HuD interacting circRNAs may compete with linear mRNAs for HuD binding and transport to synapses. Indeed, the negative correlation between the levels of *circFoxp1* and *circBrwd1* and their respective mRNAs in HuD-KO brains observed here, lends further support to this notion. Finally, as shown for *circPABPN1*, a circRNA that binds to and sequesters HuR, another mechanism of action of these RNAs is to block HuR-dependent translation of PABPN1 mRNA (Abdelmohsen et al., 2017) and other mRNAs (Li et al., 2020). Likewise, it is conceivable that HuD-bound circRNAs act as RBP sponges to regulate HuD function in the cell.

Cross-correlations analyses of circRNAs in HuD-OE and HuD-KO mice confirmed that the expression of multiple circRNAs correlate with HuD levels in the cell. These include circRNAs from genes associated with neurodevelopmental disorders and Alzheimer’s disease, e.g., *circBrwd1* and *circFoxp1* (Bacon et al., 2015; Quan et al., 2020); involved in neuronal development and regeneration, e.g., *circMap1a* (Nunez and Fischer, 1997) and, encoding members of the Plasticity Related Genes (PRGs) and membrane-associated guanylate kinases (MAGUKs) families, e.g., *circMagi1* and *circLppr4*, respectively, which control synaptic development and are linked to psychiatric disorders (Ito et al., 2012; Yu et al., 2015). Interestingly, *circFoxp1* and *circMap1a* are part of ceRNA networks involving miRNAs, such as miR-125a-5p, which was previously associated with aging (Makwana et al., 2017) and several protein-coding genes controlling neuronal polarity and cellular differentiation, such as *Camsap3* and *Ebf1* (Garel et al., 1999; Pongrakhananon et al., 2018). In addition, *circMap1a* also interacts with the mRNAs of *Myo6*, which is required for BDNF-mediated neurotransmission (Yano et al., 2006) and *Park7*, associated to Parkinson’s Disease and encoding a DJ-1 protein protecting brain cells from oxidative stress (Bose and Beal, 2016).

The compulsive drug-seeking behavior in Substance Use Disorders (SUDs) is thought to be mediated by the ability of the drug to induce molecular, cellular and circuit level maladaptations after repeated exposures. Several RBPs including HuD have been associated with addiction-related pathways (Li et al., 2008; Bryant and Yazdani, 2016; Oliver et al., 2018). HuD itself is listed in the Knowledgebase for Addiction Related Genes (KARG), further suggesting its role in drug addiction (Li et al., 2008). Also, not only are HuD and its target mRNAs increase in the NAc during cocaine CPP, but also HuD-OE mice show increased cocaine CPP compared to wild type littermates (Oliver et al., 2018). Using cocaine CPP, we found significant changes in circRNAs from genes regulating contextual conditioning, cognition, learning and memory. These included several HuD target circRNAs identified by RIP as well as a few predicted targets. Among these is *circSatb2*, which derives from the same gene as SATB2, a chromatin accessibility regulator involved in hippocampal-dependent long-term memory and cocaine conditioning (Li Y. et al., 2017). Another HuD target circRNA whose levels are altered during CPP is *circHomer1a*, which has previously been related to hippocampal homeostatic plasticity (You et al., 2015) and psychiatric disorders (Dube et al., 2019; Zimmerman et al., 2020). This circRNA derives from the gene encoding HOMER1, a protein associated with glutamatergic synapses and that is involved in drug addiction (Swanson et al., 2001; Ghasemzadeh et al., 2003; Szumlinski et al., 2004). Synaptic plasticity underlies changes in network dynamics and is considered to be the foundation of learning and memory (Ramirez and Arbuckle, 2016; Hegde, 2017). Therefore, it is not surprising that the top genes hosting the circRNAs whose levels are significantly increased or decreased after CPP are specifically involved in neuronal cell migration and neurite and dendrite outgrowth and branching.

Despite the recent discovery that circRNAs are highly expressed in neurons, particularly during synaptogenesis, the function of most circRNAs is far from understood. Overall our study demonstrates that HuD-circRNAs complexes are part of ceRNA networks, including mRNAs and miRNAs, which could be involved in fine tuning the post-transcriptional regulation of gene expression during synaptic plasticity, learning and memory and addiction.

## Data Availability Statement

The datasets presented in this study can be found in online repositories. The names of the repository/repositories and accession number(s) can be found below: https://www.ncbi.nlm.nih.gov/, Gene Expression Omnibus (GEO) database. Accession numbers: GSE153377, GSE153387, GSE153394, GSE153405, and GSE153406.

## Ethics Statement

The animal study was reviewed and approved by the University of New Mexico Health Sciences Center Institutional Animal Care and Use Committee.

## Author Contributions

MD designed and performed the experiments, analyzed the data, and wrote the manuscript together with NP-B and RO performed the behavioral experiments and collected samples. NP-B supervised the project and was in charge of the overall planning and data analysis. All authors discussed the results and contributed to the final manuscript.

## Conflict of Interest

The authors declare that the research was conducted in the absence of any commercial or financial relationships that could be construed as a potential conflict of interest.

## Footnotes

1 http://www.circbase.org/

2 https://www.qiagenbioinformatics.com/products/ingenuity-pathway-analysis

## Supplementary Material

FIGURE S1 *circUpf2* and *circCreb1* sequence analysis. Using previous published scripts (Bolognani et al., 2010) we identified HuD consensus binding motifs (shown in light blue) in *circUpf2* and *circCreb1* sequences. Sequences in green represent the circRNA junction.

FIGURE S2 *circBrwd1* ceRNA network. ceRNA analyses were conducted as previously described (Shao et al., 2018; Wang et al., 2018).

TABLE S1 List of genes encoding circRNAs that are significantly enriched in the HuD RNA immunoprecipatation (RIP) vs. control IgG IP.

TABLE S2 List of top 25 Biological networks and Diseases and Functions from genes encoding HuD-bound circRNAs.

TABLE S3 List of genes where HuD binds to both circRNAs and mRNAs.

TABLE S4 List of genes encoding circRNAs that bind to and are regulated by HuD.

TABLE S5 Competing endogenous RNA (CeRNA) networks for HuD-bound and -regulated circRNAs.

TABLE S6 Comparison of the list of differentially expressed circRNAs during CPP with those bound by HuD.

TABLE S7 List of IPA Pathways of protein coding-genes from circRNAs differentially-regulated by cocaine CPP.

TABLE S8 List of molecules in IPA Behavior Pathways of protein coding-genes from circRNAs differentially-regulated by cocaine CPP.
