# Supplementary figures and images for: HuD Binds to and Regulates Circular RNAs Derived From Neuronal Development- and Synaptic Plasticity-Associated Genes
Source: Front Genet. 2020 Aug 5;11:790. doi: 10.3389/fgene.2020.00790 (PMC7419605; doi:10.3389/fgene.2020.00790)

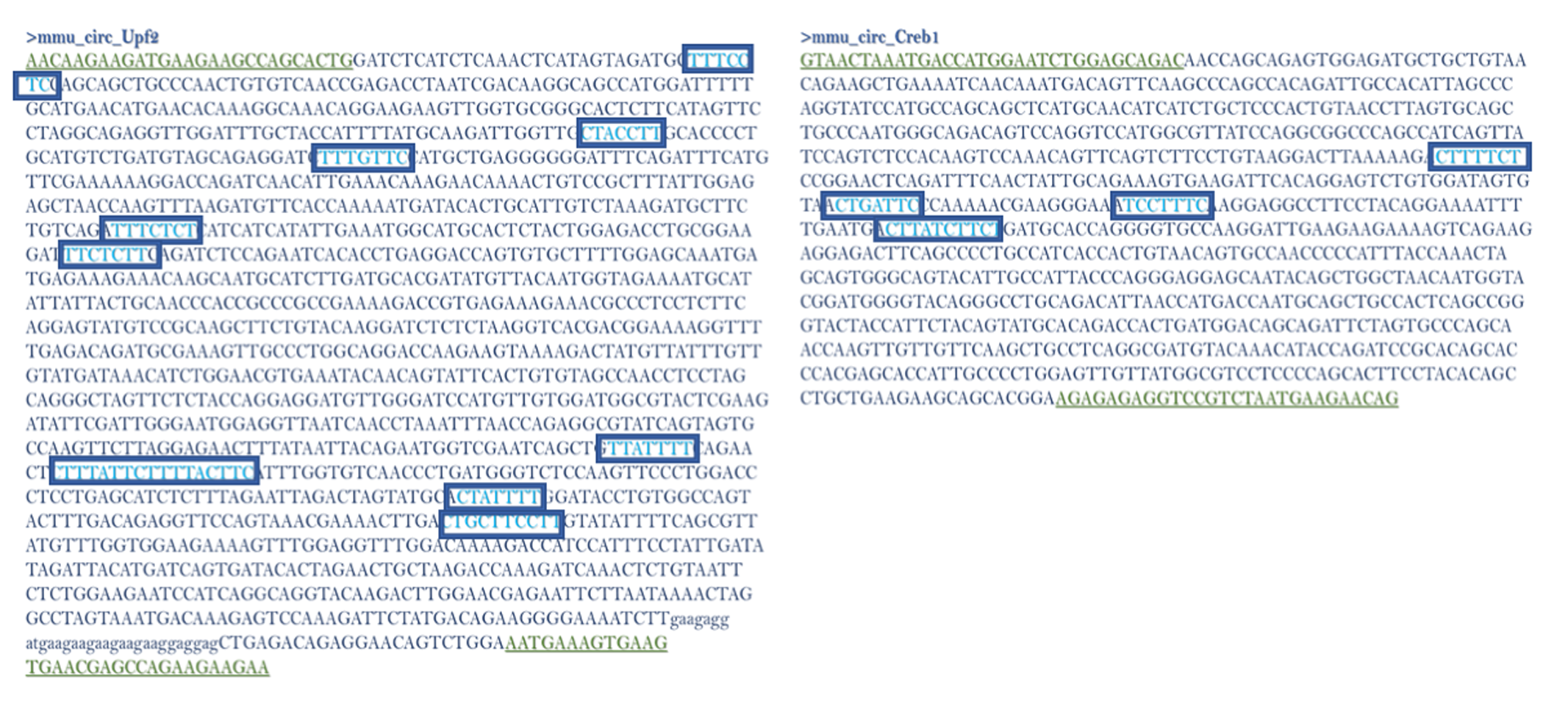

Supplement: FIGURE S1 — circUpf2 and circCreb1 sequence analysis. Using previous published scripts (Bolognani et al., 2010) we identified HuD consensus binding motifs (shown in light blue) in circUpf2 and circCreb1 sequences. Sequences in green represent the circRNA junction. [file Image_1.TIF]

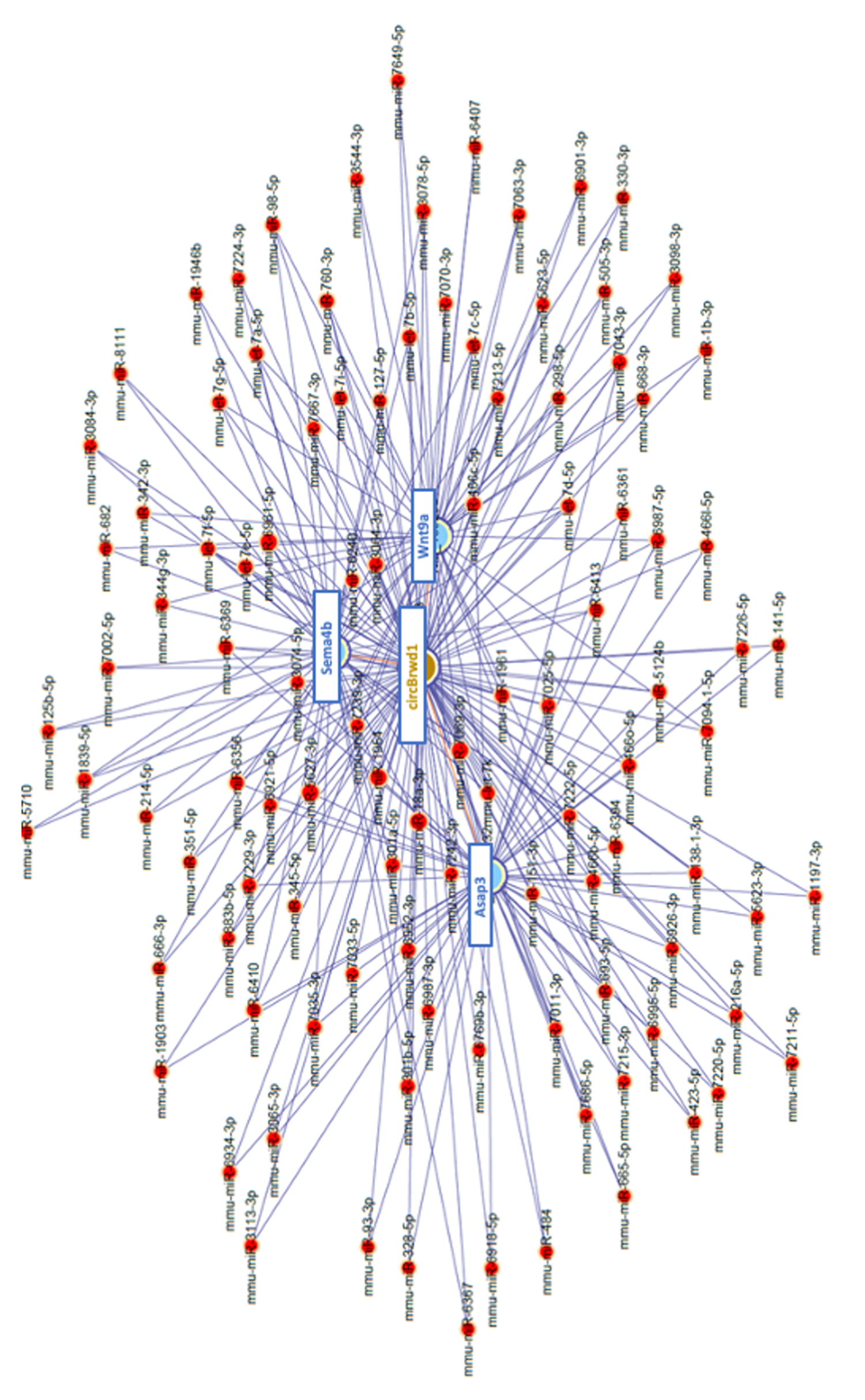

Supplement: FIGURE S2 — circBrwd1 ceRNA network. ceRNA analyses were conducted as previously described (Shao et al., 2018; Wang et al., 2018). [file Image_2.TIF]
